# Rare species disproportionally contribute to functional diversity in managed forests

**DOI:** 10.1038/s41598-022-09624-9

**Published:** 2022-04-07

**Authors:** Marco Basile

**Affiliations:** 1 Chair of Wildlife Ecology and Management, University of Freiburg, Tennenbacher Str. 4, 79106 Freiburg, Germany; 2 Swiss Federal Research Institute WSL, Zürcherstrasse 111, 8903 Birmensdorf, Switzerland; 3 Swiss Ornithological Institute, Seerose 1, 6204 Sempach, Switzerland

**Keywords:** Ecology, Environmental sciences

## Abstract

Functional diversity is linked with critical ecosystem functions, yet its relationship with numerical diversity, e.g. species richness, is not fully understood. The mechanisms linking changes of species richness, e.g. random and non-random species losses and gains, with changes of functional diversity become more relevant in the face of rapid environmental changes. In particular, non-random species changes including rare species may affect functional diversity, and the overall ecosystem function, disproportionately compared to random species changes including common species. In this study, I investigated how changes in numerical diversity of bird assemblages are related to functional diversity, and how the environment, and in particular forest management, influences such a relationship. I collected bird count data in the extensively-managed forest landscape of the Black Forest (Germany), at 82 sampling sites over three years. Data included species richness and abundance per site, and functional traits related to diet and habitat type for each species to compute functional diversity. By partitioning numerical diversity changes into five components using Price Equations, I calculated the contribution of random and non-random species losses and gains, and the abundance of common species, to functional diversity. Then I modelled these contributions as a function of several environmental variables describing broad forest conditions, and including forest management intensity. I found that, beside the major contribution of random species losses to functional diversity, non-random species losses also play a role, indicating that rare species that contribute more to functional diversity are often lost earlier than common species. The overall contribution to functional diversity of species losses is larger than that of species gains, pointing toward an ongoing simplification of the forest bird assemblage. Among all Price components, random species gains were influenced by management intensity, while other components were not influenced by any management variable. This highlight that potential conservation actions may not be effective in halting ecosystem functioning decline, as species gains do not result in increased functional diversity.

## Introduction

Biodiversity decline caused by human activities has a high potential for disrupting ecosystem processes, by affecting overall ecosystem functioning^1^. Functional diversity (FD) is a major driver of ecosystem functions, as it measures the extent of what organisms do in an ecosystem^2,3^. For this reason, FD is considered a better predictor of ecosystem functioning than species diversity^4,5^. While changes in the number of species pertain to numerical diversity of organisms, changes in the number of functional groups pertain to FD^6^. Functional groups include species that share same functional traits^7^. Measures of numerical diversity, such as species richness, may be correlated with FD, by mean of the relationship between number of species and number of functional groups in a community^2,8,9^. Henceforth, the relationship between numerical and functional diversity regulates the overall ecosystem function^10^. In general, an increase in FD is matched by an increase in species richness^11^. The relationship may vary from asymptotic in marine ecosystems^12^, to linear in arid ecosystemswith respect to precipitations^13^, or can be inferred from habitat structures in forests^14^. Generally, the relationship can change with the environmental context and the functional groups considered.

FD, like any other community measure, is the results of random and non-random assortment of species, driven by environmental filtering and biotic interactions^15–17^. While random species assortment depends on stochastic processes, non-random assortment may depend on intrinsic characteristics of a biological assemblage or community and/or on environmental conditions, and can provide insights into optimal solutions for biodiversity conservation^18–21^. For instance, non-random species loss can disproportionately affect FD, whenever a particular functional group is filtered out by environmental conditions, that can change following human alteration of ecosystems. In such cases, non-random species loss refers to the loss of species that have a high contribution to FD, compared to their occurrence rate. Consequently, in human-dominated landscapes, such as managed forests, FD may decline following non-random species loss, i.e. loss of rare species with high contribution to FD. Indeed, management can have different effects on bird species, depending on their life history traits, resulting in some species being selectively more affected than others^22–24^. Bird assemblages of managed forests often occur at lower diversity, both numerical and functional, then in primeval forests, with management type or intensity often identified as significant factors determining numerical diversity^25,26^. However, it is not clear to what extent the changes in numerical diversity of forest birds are linked to changes in the abundance of different functional groups, a scenario that could lead to the decay of forest ecosystem functioning over time.

Although it is generally understood that FD is linked with species assortment, less clear is the degree of contribution to FD that random and non-random species assortments have. Disentangling those two processes is challenging, given the concomitance of multiple confounding factors, such as historic factors, scale of the study, disturbance and species interactions^27^. To understand what is the contribution of species assortment to FD, and to pinpoint whether such contribution can be altered by human activities, can be pivotal for biodiversity conservation. Here, I used Price Equations to assess the contribution of random or non-random changes in species assortment to FD, by employing five components describing numerical changes of bird assemblages^28^. The Price components comprised: a species richness effect, related to random species losses and gains; a species composition effect, related to non-random species losses and gains; and a context dependence effect, related to between-site variation in the functional contributions of the species present, which underly the processes determining species abundance^29^. In this context, random species changes refer to the difference in functional diversity attributable to random changes in species richness. Non-random species changes, instead, refer to the difference attributable to changes in species composition. Specifically, I hypothesised that non-random species losses contribute the most to FD in human-dominated landscapes, as human activities would lead rare species with a high contribution to FD to get lost first. I further hypothesised that the contribution of non-random changes (including losses and gains) in species assortment to FD is affected by forest management. Hence, I expect that environmental variables describing management intensity will influence mainly the contribution of non-random changes to FD, rather than that of random changes. My research focused on the bird assemblages of an extensively-managed forest landscape in central Europe. I draw from previous studies investigating the relationship between biodiversity and ecosystem function with Price Equations^28,30^, and present a novel analysis that link the numerical and functional diversity of birds to ecosystem management.

## Methods

### Study area

The study was carried out in the forest landscape of the Black Forest, southwest Germany, in 82 one-hectare sites (Latitude: 47.6°- 48.3° N, Longitude: 7.7°-8.6° E; WGS 84). Sites were created within the ConFoBi research project^31^, and were located in state-owned forest at altitudes between 500 and 1400 m above sea level. Sites were at least 1 km apart and were comprised of temperate mixed mountain forest, dominated by Norway spruce (*Picea abies*), European beech (*Fagus sylvatica*) and silver fir (*Abies alba*). All sites were located in mature stands managed with single tree selection under close-to-nature forest management leading to continuous cover forests^32,33^. Close-to-nature forest management focuses on stability, productivity, diversity and continuity of forest conditions, optimizing multiple forest management goals in the same site^34^.

### Bird sampling and functional traits

Birds were sampled by employing standardized point counts with limited distance of 50 m, repeated three times/year during the period March-June 2017, 2018 and 2019, starting half an hour after sunrise with the latest end at 12:00 CET^35^. A single count lasted 20 minutes, during which every bird heard or seen was recorded. As a measure of abundance, the maximum count of individuals per site and year was used to build three matrices of sites per species abundance. Bird functional traits were retrieved from the EltonTraits 1.0 database^36^. The database included traits related to diet, forest stratum preferences for foraging, and body mass. Diet was described as percent use of different food categories, including invertebrates, mammals and birds, reptiles and amphibians, fish, unknown, scavenged items, fruits, nectar, seed. Forest stratum preferences were also described as percent use of different strata for foraging, including below water surface, on water surface, ground, understory, mid-high, canopy, aerial.

### Functional diversity analysis

To compare the different contributions of changes in species assortment to functional diversity (FD), I used a measure of FD that is independent to changes in species richness: functional dispersion^37^. Functional dispersion is the weighted mean distance of individual species to the assemblage/community weighted centroid, where weights are their relative abundances^37^. To compute it, the axis of a principal coordinate analysis from a Gower dissimilarity matrix^38^ are used as new traits. The Gower dissimilarity matrix is in turn computed from species traits and can handle both numerical and categorical data. I used functional dispersion to calculate FD using separately diet (FD_diet) and forest stratum (FD_strata) functional traits. In addition, I included also FD_comb calculated by combining the two trait sets and the average body mass. Indices were computed for every year.

### Price Equations analysis

To compare the contributions of changes in species assortment to FD, I firstly calculated the contribution of each individual species to FD. Such contribution was calculated for each year and site by multiplying single species abundances by the FD value of the same year and site, and dividing the resulting quantity by the total number of individuals across sites for each year. In the end, I obtained FD values per site for each trait set, and species contribution to FD, for each of the three study years.

Next, I calculated the contribution of random and non-random changes in species assortment to FD. My approach drew from that of Winfree et al.^30^, and employed Price Equations to partition the contribution of numerical diversity to FD among sites into five components. RICH_L and RICH_G represented the contributions of random changes (losses and gains) in species richness to FD or the species richness effect, i.e. the loss/gain of species which contribution to FD is proportional to their occurrence rate. COMP_L and COMP_G represented the contributions of non-random changes in species richness to FD or the species composition effect, i.e. the loss/gain of species which contribution to FD is disproportionate to their occurrence rate.ABUN represented the contribution of changes in the abundance of common species or the context dependence effect. Each component is calculated using Eq. (1) in Fox and Kerr^28^. So, let randomly choose two sites, a baseline and a comparison site, let $s_{c}$ be the number of species common at two sites, $s$ the species present at a baseline site, and $s^{\prime}$ those present at a comparison site. Let $z_{i}$ be the contribution to FD of species $i$ at the baseline site, and $z^{\prime}_{i}$ be the equivalent at a comparison site. And let $w_{j}^{i}$ be a species-specific variable indicating whether a species is present at both sites, the Price Equations are formulated as follows:

1 $$\begin{aligned} & \Delta FD = FD^{^{\prime}} - FD = \left( {s_{c} - s} \right)\overline{z} + \left( {s^{\prime} - s_{c} } \right)\overline{z}^{^{\prime}} + s_{c} \left( {\overline{z}^{^{\prime}} - \overline{z}} \right) = \left( {s_{c} - s} \right)\overline{z} + \left( {s^{\prime} - s_{c} } \right)\overline{z}^{^{\prime}} \\ & \; + {\text{Sp}}\left( {w_{ \cdot }^{I} ,z} \right) + \left[ {{\text{ - Sp}}\left( {w_{J}^{ \cdot } ,z^{\prime}} \right)} \right] + \mathop \sum \limits_{i = 1}^{s} \mathop \sum \limits_{j = 1}^{s^{\prime}} w_{j}^{i} \left( {z^{\prime}_{i} - z_{i} } \right) \\ \end{aligned}$$

The analytical procedure was based on repeated pairwise comparisons among sites^28^. $\Delta FD$ is the difference in functional diversity between a comparison and a baseline site, $FD^{^{\prime}} - FD$. This is equivalent to the difference between the two sites in the number of species multiplied by the mean contribution to FD per species, $\overline{z}$. The fourth part of the Eq. (1) comprises the 5 Price components. ${\text{Sp}}$ is the sum of $w_{ \cdot }^{I} = \mathop \sum \nolimits_{j = 1}^{s^{\prime}} w_{j}^{i}$ and $w_{J}^{ \cdot } = \mathop \sum \nolimits_{i = 1}^{s} w_{j}^{i}$. When species *i* = *I* and *j* = *J* are present at both sites, the variables $w_{ \cdot }^{I} = 1$ and $w_{J}^{ \cdot } = 1.$ Otherwise, they assume value 0. For further details see Fox and Kerr^28^.

Being the components additive, I also considered the cumulative random and non-random losses and gains in order to assess the contribution to FD of the overall changes in numerical diversity. By summing up RICH_L and COMP_L, and RICH_G and COMP_G, I could describe whether species gains or losses impact more on FD.

### Environmental variables

To test the hypothesis that the Price components are a function of management and to contrast the effect of management with that of the environment per se, I collected several variables at each 1-ha site. These variables described broad forest conditions, as my analysis targeted the entire bird assemblage. To assess whether changes in species assortment affected functional diversity differently in different forest conditions, I included forest management intensity, important forest structures and net primary productivity. The intensity of forest management was calculated with the Forest Management Intensity index (ForMI), which measured three different management aspects^39^: a) the proportion of harvested tree volume compared to the maximum volume; b) the proportion of non-native tree species; and c) the ratio of man-made (showing signs of cutting) vs. natural deadwood. Each component of the ForMI index span values 0–1. The cumulative index spans values 0–3, where 0 would indicate a forest not managed for timber production and 3 an intensively-managed production forest. Important forest structures included those typical of primeval or unmanaged forests^40,41^, hence more natural conditions, such as the volume of the lying deadwood or the number of standing dead trees (snags). Specifically, I used counts of snags (with diameter at breast height > 7 cm), while lying deadwood volume was collected using the line intersect method^42^. Vegetation cover, including the herb and shrub layer, was measured on eight 25-^2^ plots, systematically placed in every site. Further important forest structures included the abundance and richness of Tree-related Microhabitats (TreMs^43,44^). These microhabitats represent important structures for forest organisms^45,46,82^ and include, for instance, woodpecker cavities, trunk and mould cavities, branch holes, water-filled tree holes, insect galleries, bore holes, stem injuries and wounds (e.g., bark loss, or exposed sapwood), crown deadwood, cankers and burrs, epiphytes, nests of vertebrates and invertebrates, and can be used to characterize the forest. Finally, the Normalized Difference Vegetation Index (NDVI), which is a proxy for net primary productivity^47,48^, was computed from a satellite image retrieved from Sentinel 2A on 23 August 2016.

### Data analysis

To test whether management or environmental variables influence the Price components, I built linear mixed-effect models using the R package ‘lme4′^49^. Each model included a Price component (i.e. RICH_G, RICH_L, COMP_G, COMP_L, ABUN) as response variable and one management or environmental variable as explanatory. The random error was modelled by years and models fit using maximum likelihood, to allow model comparison^50^. The explanatory power of each model was then compared against a null model, hence a model without explanatory variables which assumes that changes in Price components follow a Gaussian distribution with a random error. The comparison made use of a Likelihood Ratio Test (LRT), which assess the goodness of fit of two competing models. A significant test indicates that one model has a superior predictive power in explaining changes in Price components. Residuals of significant models (*p* < 0.05) were checked for autocorrelation and dispersion, using the R package ‘DHARMa’^51^, and no test showed significant results, indicating that autocorrelation was not an issue and the use of a Gaussian error distribution was appropriate. All analyses were performed in R environment^52^.

## Results

**Figure 1:**
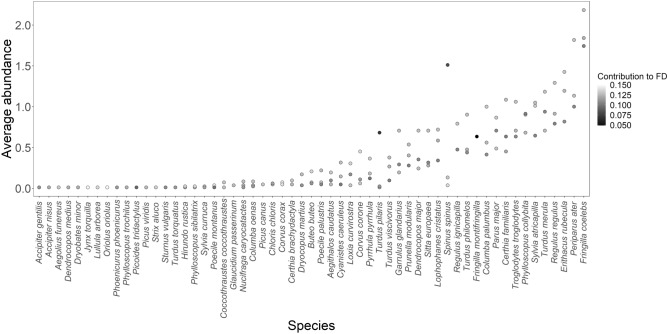
Average species abundance per year and species contribution to FD. Species are ordered along the x-axis by ascending abundance.

Over three years, I collected 4238 observations of birds belonging to 61 species. Almost half of the species (28) fed mainly on invertebrates, whereas 17 species were omnivore, 8 species had plant/seed as main diet and 8 species fed mainly on vertebrates. Regarding the preferred forest stratum, 25 species used mainly the ground stratum, 11 species were associated with the understory, for 18 species the middle-high stratum was considered the preferred one and 6 species used mainly the upper canopy. Median body mass across species was 24.26 g (range = 5.54–927.97 g). Functional diversity was similar across trait sets, ranging FD_diet = 0.06–0.19 (mean = 0.14), FD_strata = 0.04 – 0.14 (mean = 0.11), and FD_comb = 0.05 – 0.15 (mean = 0.12). Species contribution to FD averaged 0.12 ± 0.01 SD), with common species (abundance ≥ mean abundance) average contribution to FD of 0.12 ± 0.02 SD, and rare species (abundance < mean abundance) contribution to FD of 0.12 ± 0.01 SD (Fig. 1). Dietary traits of rare species differed from common species in relation to main diet based on plant/seed (7 versus 2 species) and vertebrates (8 versus 1 species). Preferred forest strata including ground and canopy were more represented among rare species compared to common ones (17 versus 9 and 6 versus 1, respectively). Rare species were on average heavier than common species (148.2 ± 230.5 g versus 81 ± 151.5 g).

**Figure 2:**
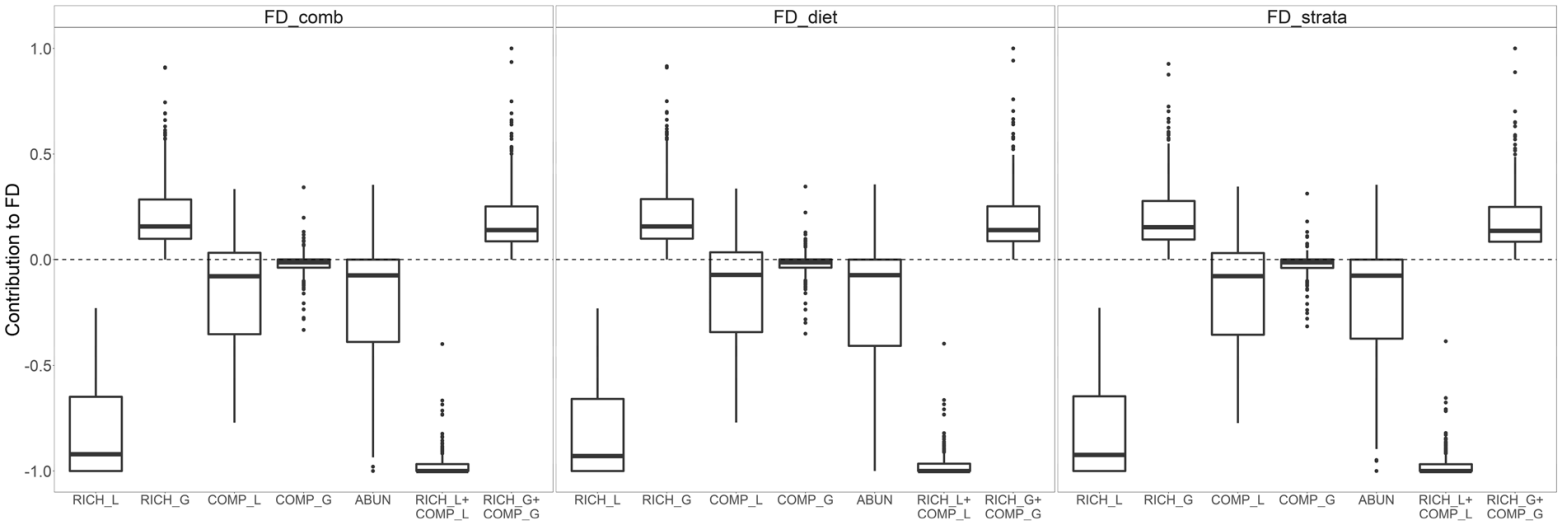
Relative contribution of the Price Equation components describing changes in species assortment, to functional diversity (FD), calculated from three different functional trait sets (FD_comb, FD_diet, FD_strata; see main text for explanation).

The results of the Price Equations were consistent across functional trait sets (Fig. 2). Random species losses (RICH_L) negatively contributed to FD, indicating that the loss of species between two random sites provides the main contribution to changes in FD. The contribution of RICH_G was similar but opposite. Non-random species losses (COMP_L) on average provided also a negative contribution to FD, indicating that species with higher contribution to FD, such as rare species, were being lost first when comparing two random sites. The contribution of COMP_G was minor and close to 0. Consequently, the cumulative contribution of random and non-random losses (RICH_L_COMP_L) to FD was strongly negative. Finally, ABUN contribution was mildly negative, indicating that changes in the abundance of common species had a mainly negative contribution to FD.

Each Price component was modelled as a function of a management or environmental variable and compared against the null model. Only few Price component models scored a significant LRT. Regarding FD_comb, the models for RICH_G scored a significant LRT when including NDVI (*χ*^*2*^ = 6.118, *p* = 0.013) and the ratio of man-made vs. natural deadwood (Idwcut) (*χ*^*2*^ = 4.022, *p* = 0.045) as covariates. The model for ABUN also scored a significant LRT when including TreM abundance (*χ*^*2*^ = 4.608, *p* = 0.032) as covariate.

Those models were significant also for FD_diet and FD_strata components. Moreover, the model of COMP_G for FD_diet also resulted significant, when the covariate was NDVI (*χ*^*2*^ = 3.846, *p* = 0.0499). Regarding FD_strata, instead, NDVI models were significant for COMP_G (*χ*^*2*^ = 4.292, *p* = 0.038) and for ABUN (*χ*^*2*^ = 3.858, *p* = 0.0495). Model diagnostics and residuals showed a good fit and a lack of autocorrelation (rho < 0.15).

**Figure 3:**
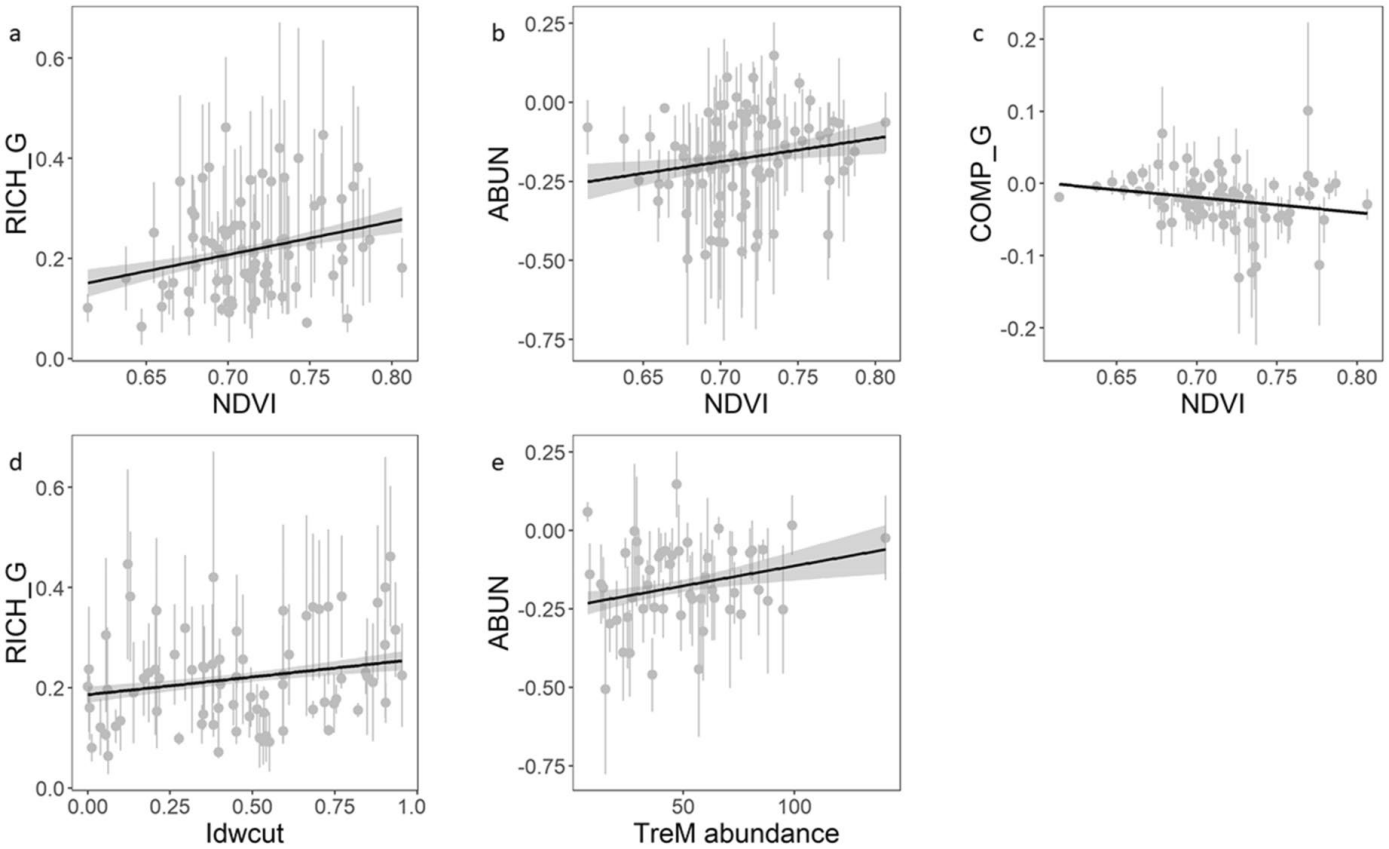
Significant effects of management and environmental variables on the Price components, describing the contribution of species assortment changes to functional diversity, resulted from linear mixed-effects models. The effects are modelled using FD_comb (**a**,**d**,**e**) and FD_strata (**b, c**) from bird assemblages across 82 forest sites. NDVI = Normalized Difference Vegetation Index; Idwcut = proportion of man-made versus natural deadwood; TreM = Tree-related Microhabitat.

RICH_G was positively influenced by NDVI (mean effect = 0.667 ± 0.268 SE) and, to a lesser extent, by Idwcut (mean effect = 0.067 ± 0.033 SE; for FD_comb analysis), indicating that the contribution of random species gains to FD increased with NDVI and the amount of man-made deadwood (Fig. 3a,d). ABUN was also positively influenced by NDVI (mean effect = 0.765 ± 0.388 SE; for FD_strata analysis) and, very slightly by TreM abundance (mean effect = 0.001 ± 0.0005 SE; for FD_comb analysis), indicating that the contribution of common species abundance to FD increased with NDVI and TreM abundance (Fig. 3b,e). Finally, COMP_G was negatively influenced by NDVI (mean effect = -0.211 ± 0.107 SE; for FD_strata analysis), indicating that the contribution of non-random species gains to FD decreased with NDVI (Fig. 3c).

## Discussion

The relationship between species diversity and the occurrence of particular functional traits in an assemblage depends on local environmental filters and biotic interactions^53^. Environmental characteristics can favour particular traits in an assemblage, determining a different species assortment than what would be expected if species were randomly distributed^54^. The existence of environmental filters acting on particular traits and associated with changes in numerical diversity has been found in several plant assemblages^55,56^. Similar results have been found for animals, such as pollinator species, where traits related with resource exploitation, such as diet breadth were non-randomly loss along a gradient of land-use intensification^19^. Among vertebrates, agriculture also seems to trigger environmental filters that decrease functional diversity of mammals and birds ^18^. The impact of human activities on functional diversity of birds is overall negative, in particular for what concerns logging activities^57^. Different kind of forest harvesting can result in different traits being lost, while species richness stays the same^58^. In order to understand and interpret potential links between numerical and functional diversity, my study attempted to quantify the contribution of changes in species assortment to FD, and understand its drivers. One of my findings is that a substantial share of bird species, that are both rare and contribute highly to FD, tends to be lost first, as indicated by the contribution of COMP_L to FD. Still, FD was mainly determined by random changes in species richness. The relationship between numerical and functional diversity is shown in many studies to be positive^11,13,59^. However, when species turnover across sites is low, as for my study area, usually functional redundancy is high and random species losses and gains may have little effect on FD^53^. In addition, the choice of the functional traits may results in diverging results, especially when comparing sites with different environmental conditions^60,61^. Considering these premises, I still found that random species loss (RICH_L) had the highest negative contribution to FD, for every functional trait set considered.

Functional diversity was overall determined by random and non-random species losses, whereas gains contributed less to FD. Considering the negative impact of human activities on FD^18,53,57^, I expected my results to reflect the extensively managed and simplified forest landscape of my study system, where a slow diversity decay is happening since decades, as for the rest of central Europe^62^. Environmental conditions can affect functional diversity and the overall ecosystem function by preventing species from establishing at sites and causing ecosystem decay over time^63,64^. However, I did not find direct evidence linking management variables with negative numerical changes underlying a decline in FD. Rather, I found that the contributions of random and non-random species gains to FD is influenced by some environmental predictors. Variables like the NDVI, the amount of man-made deadwood (Idwcut) and the amount of tree-related microhabitats can partly describe which forest conditions promote changes in species assortment that are relevant for overall functional diversity. Hence, random gains in species richness may matter more to FD in an extensively-managed forest with a high NDVI, whereas non-random gains may matter less. This may have potentially relevant consequences for forest management and for evaluating the outcome of biodiversity-oriented practices. At the same time, I would expect that management variables will have a significant influence on the contribution of species losses to FD in intensively managed forest landscapes, which usually cause a stronger decline in bird diversity^65–68^.

Human-altered environmental conditions, often acting as environmental filters, can drive functional diversity^13,20,69,70^. However, in my study system environmental/management variables can increase the importance of species gains for FD, despite species losses remain the main contributors to FD. This may be linked as well to the current situation of European forest birds, which are not experiencing harsh decline as for farmland birds^62^, but are affected by long-standing biotic homogenization^71–76^, despite the spread of close-to-nature forestry systems, such as retention forestry^77^.

This study departed from traditional approaches to modelling FD, which often provide little information about the directionality of changes in species number, focusing more on direct environmental effects on FD^78^. Instead, I partitioned the contribution of numerical diversity to FD and modelled such contributions as a function of environmental variables. This has potential relevant consequences for management actions. Conservation-oriented management actions should consider whether the removal of potential environmental filters, e.g. a change in management intensity, and the ensuing changes in species assortment will have the expected consequences in functional diversity. If not, changes in species assortment may just be the consequence of random responses to the change per se. For instance, a management action targeting tree species composition may not necessarily deliver positive functional responses^79^. An increase in broadleaf tree species would deliver higher NDVI^47,80^ and potentially affect FD, but such change may be the result of the increased contribution to FD of random species gains. Hence, the difference in FD after management may be due to changes in common species or species that contribute less to FD compared to their abundance, perhaps resulting from spill-over from neighbouring sites, rather than from improved environmental conditions^81^. In synthesis, the environmental conditions of managed forest landscapes of temperate Europe can favour random species gains and the abundance of common species, but tend not to favour many rarer species that would contribute highly to functional diversity.

## Data Availability

The data associated with this manuscript are publicly available at http://confobi-db.vm.unifreiburg.de and on Mendeley 10.17632/grv6hmd36j.1.
